# Patent Network Analysis and Quadratic Assignment Procedures to Identify the Convergence of Robot Technologies

**DOI:** 10.1371/journal.pone.0165091

**Published:** 2016-10-20

**Authors:** Woo Jin Lee, Won Kyung Lee, So Young Sohn

**Affiliations:** Department of Information and Industrial Engineering, Yonsei University, 134 Shinchon-dong, Seoul 120-749, Republic of Korea; Tianjin University, CHINA

## Abstract

Because of the remarkable developments in robotics in recent years, technological convergence has been active in this area. We focused on finding patterns of convergence within robot technology using network analysis of patents in both the USPTO and KIPO. To identify the variables that affect convergence, we used quadratic assignment procedures (QAP). From our analysis, we observed the patent network ecology related to convergence and found technologies that have great potential to converge with other robotics technologies. The results of our study are expected to contribute to setting up convergence based R&D policies for robotics, which can lead new innovation.

## 1. Introduction

The importance of robotics in modern society is increasing, because robots can be used to make everyday life more convenient as well as to promote industrial development [1]. Robot technologies are diverse enough to cover various fields such as mobility, recognition, artificial intelligence, control, sensing, batteries, software, hardware, and networking. This kind of variety accelerated the opportunities for technological convergence. Such convergence is a recent trend and can lead to innovation in robotic technologies [2, 3].

Prediction and investigation of convergence in robot technologies is crucial for enterprises and laboratories to focus on developing related technologies. Also, it can be helpful to relevant government institutions and firms, assisting them in developing effective policies to focus. Furthermore, since robot is associated with various technologies, and interact with technologies of other area, findings of this work can help to understand a larger phenomenon beyond the robot technology convergence in robotics patents.

The main purpose of this paper is to identify convergence patterns in robot technologies. We use the analysis of patent specifications which contain knowledge and information on innovative activities along with the evidence of technological convergence [4]. The two main assures have been utilized to identify technological convergence: patent citations [5, 6, 7] and patent co-classification [8], where co-occurrence of keywords of particular technology can be an evidence of co-classification of technologies. With these two approaches, research on technological convergence has mainly focused on combining more than one field, such as biotechnology (BT) with information and communication technology (ICT) [9]. Less research has been done on convergence within a specific field of technology, such as robotics.

In this paper, we assume that convergence within robotics can be observed from those patents in which there is a co-occurrence of various robot technologies. The convergence phenomenon can usually be investigated via a network-based approach because it helps understand the overall structure of the technology and also the relationship between various technologies [10, 11]. We apply patent network analysis (PNA) to identify the co-occurrence of two robot technologies in patents registered with the United States Patent and Trademark Office (USPTO) and the Korean Intellectual Property Office (KIPO), respectively, in order to understand the patent network ecologies related to convergence. The USPTO handles major patent applications from all over the world; while Korea has advanced technologies in ICT, and the majority of KIPO patents are from Korean innovators. By investigating these two patent networks, we can compare and contrast the convergence aspects of a major network in USPTO to a local network in KIPO. Moreover, we can explore how these two patent convergence networks affect each other.

Using PNA, we map the convergence network of robot technologies, find out which technologies frequently converge, and compare the technological convergence tendencies in the USPTO and KIPO. Our secondary research objective is to determine what factors influence the convergence between two robot technologies. Identification of such factors is essential to establish technological convergence strategies. Many studies have focused on convergence, but studies with a particular focus on factors related to robotic convergence have not yet been carried out using analytical methods. We use quadratic assignment procedures (QAP) to identify the significant factors associated with the convergence of robot technologies. The QAP results can help increase the synergy in convergence.

In Section 2, we present a review of the related literature. In Section 3, we describe the data used in our paper. Section 4 explains the PNA process and presents the results, while Section 5 presents the results of the QAP method. We discuss conclusions in Section 6.

## 2. Literature Review

We review the previous literature related to technological convergence, PNA, and QAP regression analysis.

### 2.1 Technological convergence

Due to the increasing interest in convergence among dissimilar technologies, many researchers have studied convergence patterns utilizing patent information. There has been an attempt to develop a convergence indicator using forward and backward citations [12]. Cho and Kim [10] also suggested the concepts of entropy and gravity as new indicators, utilizing the citation information in the USPTO database to understand technological convergence trend in printed electronics. They also found the network visualization helps obtain a holistic view of the technological convergence interaction structure. However, the citation information is only a part of capturing the convergence processes, since they also include co-classification of patents. Kim and Kim [13] identified technological convergence within different fields by investigating patent citations and co-classification of patents. Although they identified the convergence patterns between two technologies, those within a specific technology are yet investigated. Joo and Kim [9] used a multi-dimensional contingency table based on KIPO patents. The authors applied Mantel-Haenszel’s common log odds ratio to measure the relatedness of technological fields.

Although recent technological convergences have been investigated in various fields, patterns of technological convergence in robotics have not been investigated in depth. Lee and Jeong [14] worked on finding relational patterns between Korean robot technologies. Such relational patterns can be utilized to develop R&D strategies to promote convergence. However, because this previous study used keywords that occur frequently in reports on research development projects of the Korean government, it had limited potential for uncovering convergences at the level of individual technologies even though the analysis on individual technologies makes it easier to understand technological convergences. Moreover, the previous researches used the dataset gathered in 2001, and do not reflect the recent robot technologies.

### 2.2 Patent network analysis

Social network analysis (SNA) is a useful tool to illustrate the characteristics of a network [15]. The various global and local measures of the network enabled researchers to understand the different phenomena that help develop the structure of the complex network and the interactions within it. For example, Freeman [16] developed various centrality measures to characterize the role of a node in a network at the global level. These centrality measures led researchers to identify important nodes in the network. On the contrary, some local measures have also been investigated in various fields. Gao et al. [17] used a local clustering coefficient, which measures the degree of forming a cluster, to find evolving flow patterns in the oil-water flow structure. In addition, Gao et al. [18] developed multivariate complex networks for gas-liquid flow, and provided deep insights to understand its nonlinear dynamic behavior via the clustering coefficient and closeness centrality.

Patent network is one of the popular application areas of SNA. With the growing importance of intellectual property, patents have come to represent technologies [19], and various studies have focused on patent networks to understand technological trends. Yoon and Park [20] suggested constructing a patent network using text mining, i.e., by using co-occurring keywords in each patent specification. Using patent network analysis, many researchers have investigated collaboration networks of patent applicants. De Prato and Nepelski [21] showed the position of individual companies in collaboration networks, and Zheng and Cui [22] investigated global collaboration networks in nanotechnology. Guan and Shi [23] utilized the SNA to investigate transnational citation patterns. They clustered the patents using IPC information, and found small number of countries dominating the technology have similar technological patenting pattern. Guan and Zhao [24] tried to identify universities and industries collaboration in terms of nano-biopharmaceutical patents.

Like previous research using patent network analysis to investigate technology, our study applies patent network analysis to explore convergence networks of robot technology through keywords used in each technology.

### 2.3 Quadratic assignment procedure regression

To find the factors that influence the convergence network matrix of co-occurrence of patent, we applied quadratic assignment procedures (QAP) regression. For network dyadic data, it is difficult to apply OLS in the regression because this method assumes that the observations are independent and identically distributed. For instance, the nodes in the network have links, implying a potentially dependent relationship between the directly or indirectly connected nodes. Therefore, the assumption for the OLS method would not be satisfied. Instead, Krackhardt [25] suggested QAP regression, which uses nonparametric permutation. In the QAP, rows and columns of the network matrices are permuted, and correlations are obtained between independent matrices and the dependent matrix. After repeating such permutations several times, a test statistic could be derived to test the null hypothesis of the regression. Using the QAP method, the proportion of type 1 error is lower than OLS (Ordinary least squares) procedure when the degree of autocorrelation is high [25]. In our research, we need to find the relationship between the co-occurrence matrix and other independent matrices. In the technology co-occurrence network matrix, the structural autocorrelation might appear due to the classification structure of robotics technology. Therefore we used the QAP regression method. Its inferences are based on a permutation method. Rienties et al. [26] used QAP regression to find significant factors that predict the social relations of international students in a classroom, while Barnett et al. [27] used QAP to identify factors that predict the nature of web citation among universities. Cantner and Graf [28] investigated the job mobility of scientists and technological overlap between innovators by analyzing patents with QAP regression analysis. The authors found a significant association between technological overlap between innovators and job mobility. Ju and Sohn [29] applied QAP correlation to IPC co-occurrence matrix in order to analyze the technological convergence trends and patterns in the field of rare earth elements. Qiu et al. [30] used QAP correlation to find the potential correlation between technological convergence and types of author co-occurrence represented by co-authorship, author co-citation, author bibliographic coupling, words-based author coupling, and journals-based author coupling.

## 3. Methodology

**Fig 1:**
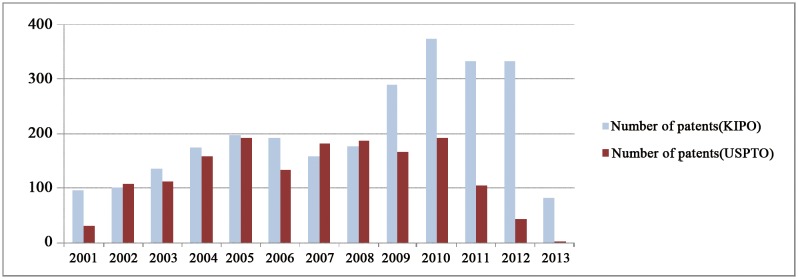
Number of patents in robot technology registered with the USPTO and KIPO.

**Fig 2:**
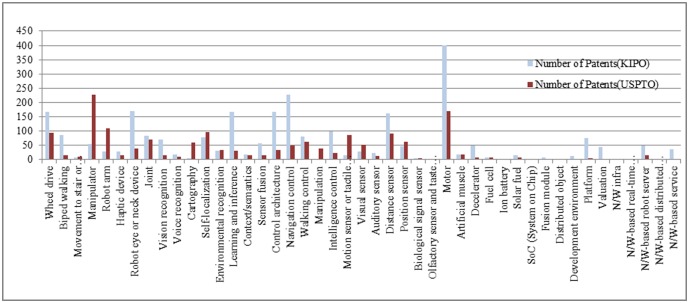
Patents in each technology area registered with the USPTO and KIPO since 2001.

In order to identify patterns of convergence among robot technologies, we use a basic-level classification of robot technology with 45 types [31]. Using target technological terms in the title, abstract, and representing claims, we gathered robotics patents registered with the USPTO and KIPO, covering from 2001 to 2013 (Fig 1). When searching those from KIPO, we used Korean and English target technological terms, and in USPTO we only used English terms. The keywords we used are illustrated in Fig 2. We observed an overall increase in the number of patents in both offices; but found a decreasing trend in the USPTO starting in 2010.

By conducting a combined search of keywords for technologies of different patent specifications, we create the co-occurrence matrix Y_Co-occur_. In this matrix, the rows and columns represent robot technologies, and the elements are the number of patents that contain the keywords related to both technologies. Appendix A shows the number of patents, number of co-occurring patents (patents that include two different technologies), and the ratio of convergence (i.e., the sum of row values of co-occurrence matrix Y_Co-occur_ divided by the total number of patents, which is converted into to a 100 percentage scale) in the KIPO and USPTO convergence networks. Mathematical formula to express Y_Co-occur_ and convergence ratio is shown below.

$$Y_{Co-occur,i,j}=Number\;of\;same\;patent\;specifications\;between\;technology_{i}\;and\;technology_{j}$$

$$Ratio\;of\;convergence_{i}\left(\%\right)=\frac{Number\;of\;converged\;patents\;in\;technology_{i}}{Number\;of\;total\;patents\;in\;technology_{i}}*100$$

To identify the role of each robotic technology in the convergence network, and to investigate the robotic technological convergence aspects of the USPTO and KIPO, we apply the PNA method to the Y_Co-occur_ matrix discussed in this section.

We calculate three centrality indices (normalized degree centrality, betweenness centrality, and eigenvector centrality) of co-occurrence to measure the role of each technology in each patent network. The degree centrality, which is the number of co-occurrences directed to different nodes, measures the tendency of a technology to converge with others. The betweenness centrality of a node measures the number of shortest paths between nodes of other pairs that pass through the node [16]; thus, a node acting as a bridge between many groups has a high betweenness centrality [32, 33]. Eigenvector centrality is the value of the first eigenvector of the matrix.

To compare the convergence networks in the USPTO and KIPO, we cluster the nodes in the network using the CONCOR (convergence of iterated correlations) method. CONCOR is a hierarchical clustering method, suggested by [34]. Since the robot technologies are a part of the entire technology, we apply the hierarchical clustering method rather than global clustering to identify clusters of robot technologies. CONCOR helps uncover the relational position or roles of the technologies in the patent network as it is based on structural equivalence. CONCOR begins by forming a square matrix of product-moment correlations between the columns of the original data. After subsequent iteration, matrix converges into a blocked form. This form is used as the basis of hierarchical clustering.

To investigate how the aforementioned Y_Co-occur_ matrix is related to the patent properties of each technology, we used QAP regression, where Y_Co-occur_ is used as a dependent variable. Using the patent attributes database, we made matrices and used them as independent variables for the QAP regression. The technology matrix with respect to IPC (International Patent Classification) overlap is X_IPC_, in which the rows represent technologies, the columns are IPC codes, and the matrix values are the number of technology patents classified into particular IPCs. We considered X_IPC_ as an independent matrix to investigate how co-classification of patents relates to convergence. We also considered the technology matrix with respect to overlap of innovators, X_Innovator_, in which the rows represent technologies, the columns are patent innovators, and the values of the matrix are the number of technology patents whose applicants were row’s innovators. The matrix of “overlap of innovators” was considered as an independent matrix to investigate the association with the technological convergence of those innovators who have advanced technologies in various robot technologies. We transformed X_IPC_ and X_Innovator_ into one-mode matrices where rows and columns are technologies by multiplying their transposed matrices [28].

Additional attribute dataset is used, in which the rows are robotic technologies and the columns represent the following various measures: the average number of patent claims of a technology, X_Avg number_, the period between the application and registration dates, X_Period_, the percentage of applicants having the most common nationalities (EU, JP, KR, US), X_EU,_ X_JP,_ X_KR,_ X_US,_ and the three aforementioned centrality measures, X_Degree,_ X_Betweenness,_ X_Eigenvector_. Investigation of the nationalities of applicants can be used for international patent strategies [35], and the proportions of nationalities related to each technology are indicated in Appendix C. The centrality measures in each technology from the patent network are calculated as shown in Appendix B. The period between a patent application date and its registration date can be used as an index of its complexity [36], which is depicted in Appendix D. The matrices in Appendices B, C, and D were converted to a matrix dataset by calculating the absolute difference and the sum of the values to find the relationship with the convergence matrix. For instance, the ij^th^ cell of the absolute difference of degree contains the absolute difference of the value of the degree of the i^th^ technology and the degree of the j^th^ technology.

We conduct a QAP regression for the dependent variable matrix Y_Co-occur_, obtained from the USPTO, against the independent variable matrices described earlier. The dependent variable matrix from co-occurrence relationship in KIPO is also used for the QAP regression with the same independent variable matrices in the earlier case.

## 4. Results

### 4.1 Data

Fig 2 shows the number of patents registered in each area of robot technology. The USPTO had a large number of patents in the areas of “manipulator,” “motor,” “robot arm,” and “self-localization,” whereas the KIPO had the most patents in “motor,” “navigation control,” “control architecture,” and “robot eye or neck device.” Counting both offices, “motor” technology had the most patents, indicating that “motor” is the main robot technology overall. However, the two offices differed in their other major technologies as shown above.

### 4.2 Co-occurrence matrix and centrality measures

Table 1 lists the technologies with convergence ratios greater than 100, among those with at least ten patents. This helps to filter the technologies that have only a few patents, which can cause overestimation of technology convergence ratio.

**Table 1:** Technologies with ratio of convergence greater than 100%.

USPTO	Ratio (%)	KIPO	Ratio (%)
Vision recognition	187	Visual sensor	232
Sensor fusion	129	Auditory sensor	227
Biped walking	121	Motion sensor or tactile sensor	225
Motion sensor or tactile sensor	109	Haptic device	159
Visual sensor	108	Walking control	136
Wheel drive	105	Joint	134
Intelligence control	105	N/W-based service	132
Environmental recognition	103	Biped walking	121
		Artificial muscle	117
		Intelligence control	115
		N/W-based robot server	108
		Decelerator	102
Average of convergence ratio	65	Average of convergence ratio	84

The technology areas of “biped walking,” “motion sensor or tactile sensor,” “visual sensor,” and “intelligence control” showed convergence ratios greater than 100% for both patent offices. The average convergence ratio among the patents studied was 65% in the USPTO and 84% in the KIPO, meaning that the KIPO patents had a greater tendency toward convergence.

The technologies that have the top 10 highest centrality measures in the USPTO and KIPO are shown in Table 2.

**Table 2:** Top 10 robotic technologies for each centrality measure.

USPTO			KIPO		
Normalized degree centrality	Betweenness centrality	Eigenvector centrality	Normalized degree centrality	Betweenness centrality	Eigenvector centrality
Motion sensor or tactile sensor	Motor	Motion sensor or tactile sensor	Motor	Intelligence control	Motor
Motor	Motion sensor or tactile sensor	Motor	Navigation control	Platform	Wheel drive
Manipulator	Cartography	Distance sensor	Wheel drive	Joint	Navigation control
Self-localization	Distance sensor	Position sensor	Control architecture	Navigation control	Control architecture
Position sensor	Joint	Wheel drive	Distance sensor	Control architecture	Distance sensor
Distance sensor	Manipulator	Manipulator	Robot eye or neck device	N/W-based robot server	Robot eye or neck device
Visual sensor	Robot eye or neck device	Visual sensor	Intelligence control	Motor	Biped walking
Wheel drive	Self-localization	Self-localization	Joint	Wheel drive	Walking control
Cartography	Control architecture	Cartography	Walking control	Distance sensor	Intelligence control
Navigation control	Intelligence control	Navigation control	Biped walking	N/W-based service	Joint

According to the normalized degree centrality results, the technology areas of “motor,” “distance sensor” and “wheel drive” had the highest values. For this measure, the “motion sensor or tactile sensor” technology differed the most between the two offices, with the USPTO value being the highest (13.2) and the KIPO value (1.5) being very low.

Nodes that have high betweenness centrality show a high probability of convergence in the future. In addition, this measure is an indicator that shows the linkage of all technologies in the field of robotics. The “motor,” “distance sensor,” “joint,” “control architecture,” and “intelligence control” technologies have high betweenness values in the registered patents of both offices. These nodes link the convergences of other technologies. The “joint,” “control architecture,” and “intelligence control” technologies have low degree centralities but high betweenness centralities, implying that they do not directly co-occur with other technologies often, but do help other nodes to converge.

**Fig 3:**
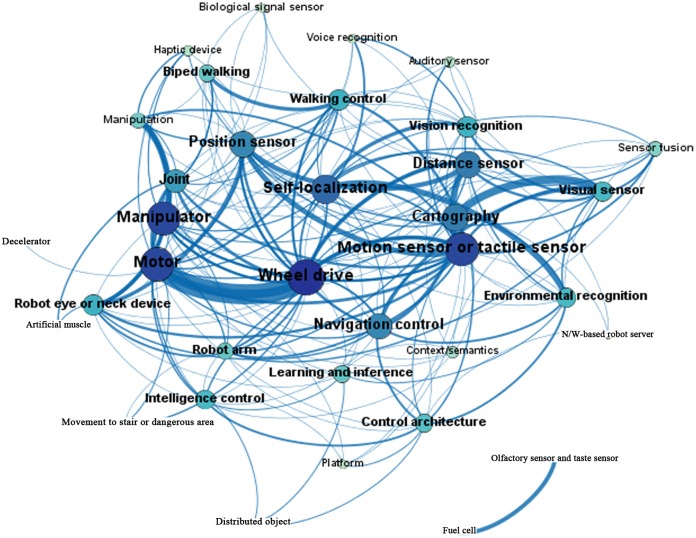
USPTO patent network map.

**Fig 4:**
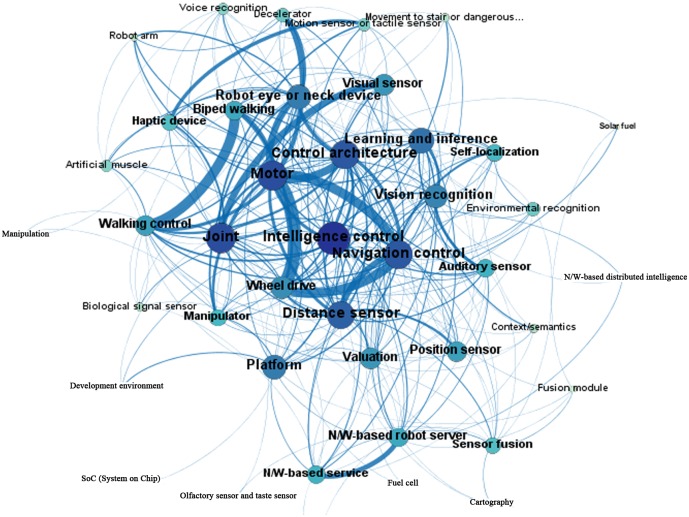
KIPO patent network map.

Eigenvector centrality incorporates the weight of each tie and indicates the influence of a node in the network, including its frequency in paths [37]. By this measure, the technology areas of “motor,” “wheel drive,” and “distance sensor” have a high degree of influence in the global network. They can be interpreted as core technologies in the convergence network [38]. Figs 3 and 4 visualize the co-occurrence networks of the patents that are registered by the USPTO and KIPO, with technologies as the nodes and the number of co-occurrence patents as the ties between nodes. In Figs 3 and 4, the size of each node is proportional to its node degree, and the thickness of each path is proportional to its strength.

According to the USPTO patent network, the two strongest relationships are between “cartography” and “visual sensor”, and between “motor” and “wheel drive.” In contrast, the two strongest relationships in the KIPO network are between “walking control” and “biped walking” and between “motor” and “wheel drive.”

### 4.3 CONCOR

Table 3 shows the result of the CONCOR. We can find the USPTO’s patents are grouped into five clusters and KIPO’s are eight. The same group of the CONCOR result means that it has similar convergence patterns and roles in the patent network.

**Table 3:** CONCOR result from both patent offices.

Technology-USPTO	Cluster	Technology-KIPO	Cluster
Wheel drive	1	Wheel drive	1
Manipulator	1	Biped walking	1
Movement to stair or dangerous area	1	Manipulator	1
Haptic device	1	Robot arm	1
Joint	1	Robot eye or neck device	1
Walking control	1	Control architecture	1
Manipulation	1	Walking control	1
Intelligence control	1	Intelligence control	1
Decelerator	1	Decelerator	1
Biped walking	2	Platform	1
Robot arm	2	Movement to stair or dangerous area	2
Robot eye or neck device	2	Self-localization	2
Motion sensor or tactile sensor	2	Navigation control	2
Distance sensor	2	Distance sensor	2
Position sensor	2	Position sensor	2
Biological signal sensor	2	Motor	2
Motor	2	Fuel cell	2
Artificial muscle	2	Solar fuel	2
Vision recognition	3	Haptic device	3
Voice recognition	3	Joint	3
Cartography	3	Motion sensor or tactile sensor	3
Self-localization	3	Visual sensor	3
Environmental recognition	3	Voice recognition	4
Sensor fusion	3	Manipulation	4
Navigation control	3	Artificial muscle	4
Visual sensor	3	Vision recognition	5
Auditory sensor	3	Environmental recognition	5
Learning and inference	4	Learning and inference	5
Context/semantics	4	Context/semantics	5
Control architecture	4	Auditory sensor	5
Platform	4	Valuation	5
N/W-based robot server	4	Sensor fusion	6
Olfactory sensor and taste sensor	5	Biological signal sensor	6
Fuel cell	5	N/W infra	6
Ion battery	5	N/W-based robot server	6
Solar fuel	5	N/W-based distributed intelligence	6
SoC (System on Chip)	5	N/W-based service	6
Fusion module	5	Cartography	7
Distributed object	5	Olfactory sensor and taste sensor	7
Development environment	5	SoC (System on Chip)	7
Valuation	5	Fusion module	7
N/W infra	5	Development environment	7
N/W-based real-time distributed control	5	Ion battery	8
N/W-based distributed intelligence	5	Distributed object	8
N/W-based service	5	N/W-based real-time distributed control	8

In both USPTO and KIPO, the cluster 1 represents the technology related to the movement of the robots. In USPTO the technology related to sensors are in the cluster 3, but in KIPO it is further subdivided to cluster 3 and 4. In USPTO, both fuel and network technologies are in the same cluster, while the fuel technology in KIPO is in the same cluster with the sensor technologies.

### 4.4 QAP

QAP analysis was conducted by utilizing the USPTO and KIPO patent co-occurrence data. The analysis results are shown in Tables 4 and 5, and Appendices E and F. First, we used the USPTO co-occurrence data as the dependent variable matrix. The rows and columns of the dependent variable matrix were permuted 2,000 times. The independent variable matrices related to USPTO or KIPO were used for the QAP analysis, and the adjusted R^2^ of the QAP regression analysis was 0.485. The significant independent variable matrices are shown in Table 4.

**Table 4:** Significant results of the QAP regression.

Source of Independent Matrix	Independent Matrix	Standardized Coefficient	Standardized Error	P-value
USPTO	Absolute difference of degree centralities	0.52***	0.1045	0.0005
USPTO	Absolute difference of eigenvector centralities	-0.73***	0.0209	0.0005
USPTO	Sum of eigenvector centralities	0.52***	0.0159	0.0005
USPTO	Absolute difference of percentages of innovators of JP nationality	0.09*	0.6108	0.0585
USPTO	Sum of percentages of innovators of JP nationality	-0.17*	1.2764	0.0965
USPTO	Absolute difference of percentages of innovators of US nationality	-0.11**	0.5395	0.0270
USPTO	Overlap in innovators	0.38**	0.0045	0.0005
USPTO	Absolute difference of periods between application and registration dates	-0.28**	0.0014	0.0380
USPTO	Sum of periods between application and registration dates	0.29**	0.0014	0.0340
USPTO	Absolute difference of numbers of claims	-0.06*	0.0237	0.0860
KIPO	Sum of degree centralities	-0.7*	0.4936	0.0670
KIPO	Absolute difference of betweenness centralities	-0.11**	0.0554	0.0365
KIPO	Absolute difference of eigenvector centralities	-0.21*	0.0167	0.0700
KIPO	Sum of eigenvector centralities	0.47**	0.0307	0.0350
KIPO	Absolute difference of percentages of innovators of EU nationality	-0.06*	5.9121	0.0975
KIPO	Sum of percentages of innovators of EU nationality	-0.03**	0.2936	0.0440
KIPO	Sum of percentages of innovators of JP nationality	-0.11*	4.0378	0.0720
KIPO	Absolute difference of periods between application and registration dates	-0.07*	0.0026	0.0730
KIPO	Sum of periods between application and registration dates	-0.16**	0.0035	0.0155

(dependent matrix = USPTO Y_Co-occur_)

* p<0.1

** p<0.05

*** p<0.01

**Table 5:** Significant results of QAP regression.

Source of Independent Matrix	Independent Matrix	Standardized Coefficient	Standard Error	P-value
USPTO	Absolute difference of percentages of innovators of EU nationality	0.03***	0.1	0.001
USPTO	Sum of percentages of innovators of EU nationality	0.03***	0.02	0.001
USPTO	Absolute difference of percentages of innovators of JP nationality	0.08**	0.02	0.001
USPTO	Absolute difference of percentages of innovators of US nationality	-0.06*	0.61	0.059
USPTO	Sum of percentages of innovators of US nationality	0.14*	1.28	0.097
USPTO	Overlap in IPC	-0.06*	0.54	0.027
KIPO	Absolute difference of degree centralities	0.45***	0	0.001
KIPO	Absolute difference of eigenvector centralities	-1.04***	0	0.034
KIPO	Sum of eigenvector centralities	1.18***	0.02	0.086
KIPO	Absolute difference of percentages of innovators of JP nationality	-0.33***	0.49	0.067
KIPO	Absolute difference of percentages of innovators of KR nationality	0.24***	0.14	0.066
KIPO	Absolute difference of percentages of innovators of US nationality	-0.2***	0.18	0.045
KIPO	Sum of percentages of innovators of US nationality	0.21***	0.06	0.037
KIPO	Overlap in innovators	0.41***	0.02	0.07
KIPO	Absolute difference of number of claims	-0.18***	0.03	0.035
KIPO	Sum of number of claims	0.26***	5.91	0.098

(Dependent matrix: KIPO co-occurrence matrix)

* p<0.1

** p<0.05

*** p<0.01

In detail, the “absolute difference of degree centralities (USPTO)”, a difference of the convergence tendency between two technologies was positively significant. In addition, as the eigenvector centrality means the strength of node’s influence in the network, the negative coefficient in the “absolute difference of eigenvector centralities (USPTO)” and the positive coefficient in the “sum of eigenvector centralities (USPTO)” imply that highly influential technologies tend to converge with each other.

Moreover, some other variables were also significantly related to USPTO co-occurrence. The tendency to converge increases as the number of technologies shared by the same innovators increases, called “overlap in innovators (USPTO)”. In addition, the results for the “absolute difference of periods between application and registration dates (USPTO)” and “sum of the period for application and that for registration (USPTO)” imply that technologies for which patent registration evaluation takes a long time seem to converge with other technologies having the same property. This is because patents that include multiple technologies are more complex and thus require more time for the patent office to reach a decision about whether to register them.

The one of matrices related to KIPO convergence network centrality, “sum of eigenvector centralities (KIPO),” had a positive relationship with USPTO co-occurrence. In contrast, three other centrality matrices, the “sum of degree centralities (KIPO),” “absolute difference of betweenness centralities (KIPO),” and “absolute difference of eigenvector centralities (KIPO),” had negative relationships. This shows that the centrality measures in the KIPO patent network have a relationship with the USPTO.

Second, we regressed the KIPO co-occurrence matrix onto the independent variable matrices. The dependent variable matrix was permuted 2,000 times, and the adjusted R^2^ of the QAP regression was 0.685. The significant independent variable matrices and the coefficients are displayed in Table 5.

Seventeen matrices showed statistically significant relationships with the KIPO co-occurrence matrix. The “absolute difference of degree centralities (KIPO),” “absolute difference of eigenvector centralities (KIPO),” and “sum of eigenvector centralities (KIPO)” were similar to those of the USPTO. The “absolute difference of percentages of innovators of U.S. nationality (KIPO)” and “sum of percentages of innovators of U.S. nationality (KIPO)” revealed that technologies with high ratios of U.S. innovators tended to converge. In addition, the “overlap in innovators (KIPO)” also had an effect on convergence. Unlike the relationship between KIPO centrality and USPTO co-occurrence, the innovator nationality percentages of USPTO patents had statistically significant effects; these were the “absolute difference of percentages of innovators of EU nationality (USPTO),” “sum of percentages of innovators of EU nationality (USPTO),” “absolute difference of percentages of innovators of JP nationality (USPTO),” “absolute difference of percentages of innovators of U.S. nationality (USPTO),” and “sum of percentages of innovators of U.S. nationality (USPTO).”

## 5. Conclusion

In this study, we investigated robotics patents that had been registered with the KIPO and the USPTO, respectively, to find convergence patterns among 45 robotics technologies. By investigating the number of patents in various technology areas, we found that the two offices differed in terms of the technologies they patented the most. They commonly patented “motor” most, but the USPTO showed specializations in “manipulator,” “wheel drive,” and “motion sensor or tactile sensor,” whereas the KIPO had many patents in “joint,” “navigation control,” and “intelligence control.”

Second, in terms of convergence, we found that the USPTO leads in the visual and recognition areas (vision recognition, robot eye or neck device, sensor fusion, environmental recognition) as they have the top highest weighted links in the patent network. In contrast, the KIPO leads in areas related to robotic movement (movement to stair or dangerous area, walking control, joint, biped walking). This contributes to understanding how technology convergence in robotics differs between two patent offices.

Furthermore, the average ratio of convergence indicated that KIPO patents generally have a stronger tendency toward convergence and the application of convergence technologies. This implies that Korean development of robot technology is focused on new robotic products using convergence of a variety of related technologies, assuming that most of the innovators of KIPO patents are Korean. In contrast robot in US technology market is focused on fundamental technologies.

Third, using PNA, we calculated three measurements of centrality for each technology in the patent convergence network. This information can be used to identify the central technologies in the field of robotics. For the USPTO, “motion sensor or tactile sensor” showed high centrality measures, whereas for the KIPO, “wheel drive” did so. In both patent offices, “motor” technology was high in the three centrality measures. “Motor” technology is a basic technology for various robot patents and has wide externality.

Fourth, we used QAP to identify factors that had statistically significant effects on technological convergence patterns. The QAP results revealed that the KIPO centrality matrix has a significant relationship with the USPTO patent convergence network. In contrast, the centrality measures of the USPTO had less influence on those of the KIPO. This can be interpreted as meaning that many KIPO patents are also co-registered in the USPTO, so that the centrality of KIPO influences the USPTO. In contrast, few patents registered with the USPTO are also registered with the KIPO. This shows that, because the U.S. is the major market for robotics, KIPO innovators who applied for patents attempt to register with the USPTO; because the Korean market is small, however, international innovators do not try to register patents with the KIPO.

The QAP results we conducted can be put to practical use. Understanding the factors that have relationships with convergence can be useful in establishing R&D polices at the levels of both government and firms. For example, because the QAP results indicated that overlap of innovators has a relationship with convergence, policies to promote multinational co-work can be implemented to develop patents dealing with convergent robot technology. On the other hand, we can predict the future through the identified convergence pattern in the QAP results. We have identified that the frequently converged technologies are willing to converge with those that are rarely converged, since the difference in degree centrality in the patent network was positively correlated with the technological convergence. In addition, the influential technologies tend to be converged with each other based on the QAP results, the negative correlation of the difference in eigenvector centralities, and the positive correlation of the sum of eigenvector centralities.

This research contributes to the understanding of technological convergence in robotics, but it also has limitations. The main limitation of our research is that we did not consider patent quality. Backward citations are generally used to measure patent quality, but our database did not provide citation information. By considering patent valuation [39], we could have clearly determined the direct relationships between technological convergence and the development of high-value patents. We plan to use this methodology in a future paper and to compare and analyze the Japan Patent Office, Chinese State Intellectual Property Office, European Patent Office, and other major patent offices. We also plan to analyze patent convergence in other technological fields. Future work may also include studying the changes in convergence patterns over time.

## Supporting Information

S1 Appendix Appendices for this manuscript. (A) Convergence ratios for each technology. (B) centrality measures of each technology. (C) proportions of innovator nationalities for each technology. (D) average interval between application and registration dates and average number of claims for each technology. (E) QAP results: USPTO co-occurrence matrix and coefficient of independent variables. (F) QAP results: KIPO co-occurrence matrix and coefficient of independent variables.
(DOCX)
