# Supplementary material for: Patent Network Analysis and Quadratic Assignment Procedures to Identify the Convergence of Robot Technologies
Source: PLoS One. 2016 Oct 20;11(10):e0165091. doi: 10.1371/journal.pone.0165091 (PMC5072598; doi:10.1371/journal.pone.0165091)
Supplement: S1 Appendix — (A) Convergence ratios for each technology. (B) centrality measures of each technology. (C) proportions of innovator nationalities for each technology. (D) average interval between application and registration dates and average number of claims for each technology. (E) QAP results: USPTO co-occurrence matrix and coefficient of independent variables. (F) QAP results: KIPO co-occurrence matrix and coefficient of independent variables. (DOCX) [file pone.0165091.s001.docx]

**Appendix**

Appendix A. Convergence ratios for each technology

| **Technologies** | **USPTO** | | | **KIPO** | | |
| --- | --- | --- | --- | --- | --- | --- |
|  | **Number of patents** | **Number of co-occurring patents** | **Convergence ratio** | **Number of patents** | **Number of co-occurring patents** | **Convergence ratio** |
| Wheel drive | 94 | 99 | 105 | 168 | 159 | 95 |
| Biped walking | 14 | 17 | 121 | 86 | 104 | 121 |
| Movement to stair or dangerous area | 9 | 6 | 67 | 8 | 13 | 163 |
| Manipulator | 228 | 48 | 21 | 55 | 47 | 85 |
| Robot arm | 109 | 30 | 28 | 28 | 21 | 75 |
| Haptic device | 15 | 10 | 67 | 29 | 46 | 159 |
| Robot eye or neck device | 40 | 36 | 90 | 169 | 117 | 69 |
| Joint | 69 | 32 | 46 | 82 | 110 | 134 |
| Vision recognition | 15 | 28 | 187 | 71 | 68 | 96 |
| Voice recognition | 11 | 10 | 91 | 17 | 15 | 88 |
| Cartography | 60 | 48 | 80 | 5 | 4 | 80 |
| Self-localization | 97 | 60 | 62 | 77 | 39 | 51 |
| Environmental recognition | 34 | 35 | 103 | 31 | 18 | 58 |
| Learning and inference | 30 | 14 | 47 | 166 | 70 | 42 |
| Context/semantics | 14 | 7 | 50 | 18 | 11 | 61 |
| Sensor fusion | 14 | 18 | 129 | 56 | 21 | 38 |
| Control architecture | 33 | 27 | 82 | 166 | 145 | 87 |
| Navigation control | 49 | 45 | 92 | 226 | 191 | 85 |
| Walking control | 63 | 32 | 51 | 81 | 110 | 36 |
| Manipulation | 39 | 29 | 74 | 3 | 3 | 100 |
| Intelligence control | 22 | 23 | 105 | 99 | 114 | 115 |
| Motion sensor or tactile sensor | 85 | 93 | 109 | 16 | 36 | 225 |
| Visual sensor | 51 | 55 | 108 | 28 | 65 | 232 |
| Auditory sensor | 12 | 8 | 67 | 22 | 50 | 227 |
| Distance sensor | 92 | 55 | 60 | 161 | 128 | 80 |
| Position sensor | 61 | 56 | 92 | 47 | 32 | 68 |
| Biological signal sensor | 4 | 4 | 100 | 5 | 9 | 180 |
| Olfactory sensor and taste sensor | 1 | 8 | 800 | 3 | 2 | 67 |
| Motor | 170 | 73 | 43 | 400 | 293 | 73 |
| Artificial muscle | 19 | 5 | 26 | 18 | 21 | 117 |
| Decelerator | 7 | 1 | 14 | 48 | 49 | 102 |
| Fuel cell | 8 | 8 | 100 | 6 | 2 | 33 |
| Ion battery | 0 | 0 | 0 | 0 | 0 | 0 |
| Solar fuel | 7 | 0 | 0 | 15 | 5 | 33 |
| SoC (System on Chip) | 0 | 0 | 0 | 1 | 1 | 100 |
| Fusion module | 0 | 0 | 0 | 6 | 6 | 100 |
| Distributed object | 2 | 6 | 300 | 1 | 0 | 0 |
| Development environment | 1 | 0 | 0 | 13 | 7 | 54 |
| Platform | 4 | 6 | 150 | 76 | 50 | 66 |
| Valuation | 0 | 0 | 0 | 43 | 38 | 88 |
| N/W infra | 0 | 0 | 0 | 1 | 2 | 200 |
| N/W-based real-time distributed control | 0 | 0 | 0 | 0 | 0 | 0 |
| N/W-based robot server | 16 | 3 | 0 | 50 | 54 | 108 |
| N/W-based distributed intelligence | 0 | 0 | 0 | 2 | 5 | 250 |
| N/W-based service | 0 | 0 | 0 | 37 | 49 | 132 |

Appendix B. Centrality measures of each technology

| **Technology** | **USPTO** | | | **KIPO** | | |
| --- | --- | --- | --- | --- | --- | --- |
|  | **Normalized degree centrality** | **Betweenness centrality** | **Eigenvector centrality** | **Normalized degree centrality** | **Betweenness centrality** | **Eigenvector centrality** |
| Wheel drive | 7.53 | 1.44 | 40.53 | 6.57 | 3.75 | 58.42 |
| Biped walking | 2.27 | 0.11 | 8.86 | 4.3 | 1.01 | 29.81 |
| Movement to stair or dangerous area | 0.85 | 0 | 4.91 | 0.54 | 0 | 4.85 |
| Manipulator | 8.52 | 2.5 | 36.73 | 1.94 | 1.47 | 13.75 |
| Robot arm | 4.26 | 0.82 | 22.88 | 0.87 | 0.05 | 6.32 |
| Haptic device | 1.42 | 0.12 | 7.23 | 1.9 | 1.44 | 6.15 |
| Robot eye or neck device | 5.11 | 2.27 | 25.53 | 4.83 | 1.77 | 30.05 |
| Joint | 4.55 | 2.67 | 21.08 | 4.55 | 6.33 | 19.54 |
| Vision recognition | 3.98 | 0.92 | 16.41 | 2.81 | 2.14 | 8.44 |
| Voice recognition | 1.42 | 0.03 | 5.94 | 0.62 | 0.2 | 3.13 |
| Cartography | 6.82 | 3.41 | 29.39 | 0.17 | 0 | 0.31 |
| Self-localization | 8.52 | 2.25 | 33.96 | 1.61 | 0.4 | 9.28 |
| Environmental recognition | 4.97 | 0.46 | 20.01 | 0.74 | 0.11 | 3.14 |
| Learning and inference | 1.99 | 1.16 | 4.99 | 2.89 | 1.82 | 10.06 |
| Context/semantics | 0.99 | 0.24 | 3.74 | 0.45 | 0.02 | 0.82 |
| Sensor fusion | 2.56 | 0.05 | 14.1 | 0.87 | 2.39 | 2.66 |
| Control architecture | 3.84 | 2.19 | 11.86 | 5.99 | 5.33 | 40.72 |
| Navigation control | 6.39 | 1.34 | 28.81 | 7.89 | 5.53 | 56.44 |
| Walking control | 4.55 | 0.46 | 20.49 | 4.55 | 2.47 | 29.4 |
| Manipulation | 4.12 | 0.2 | 20.22 | 0.12 | 0.02 | 0.37 |
| Intelligence control | 3.27 | 1.74 | 12.18 | 4.71 | 7.91 | 24.42 |
| Motion sensor or tactile sensor | 13.21 | 3.81 | 57.68 | 1.49 | 0.22 | 4.03 |
| Visual sensor | 7.81 | 0.63 | 34.49 | 2.69 | 0.59 | 13.47 |
| Auditory sensor | 1.14 | 0.15 | 6.79 | 2.07 | 0.35 | 9.43 |
| Distance sensor | 7.81 | 2.99 | 42.52 | 5.29 | 3.45 | 31.31 |
| Position sensor | 7.95 | 1.29 | 42.4 | 1.32 | 0.88 | 7.05 |
| Biological signal sensor | 0.57 | 0 | 2.96 | 0.37 | 0.23 | 1.67 |
| Olfactory sensor and taste sensor | 1.14 | 0 | 0 | 0.08 | 0.01 | 0.17 |
| Motor | 10.37 | 8.26 | 46.1 | 12.11 | 3.81 | 75.47 |
| Artificial muscle | 0.71 | 0.01 | 2.37 | 0.87 | 0.42 | 4.15 |
| Decelerator | 0.14 | 0 | 0.96 | 2.02 | 0.39 | 17.59 |
| Fuel cell | 1.14 | 0 | 0 | 0.08 | 0 | 0.86 |
| Ion battery | 0 | 0 | 0 | 0 | 0 | 0 |
| Solar fuel | 0 | 0 | 0 | 0.21 | 0.08 | 1.62 |
| SoC (System on Chip) | 0 | 0 | 0 | 0.04 | 0 | 0.08 |
| Fusion module | 0 | 0 | 0 | 0.25 | 0.34 | 0.6 |
| Distributed object | 0.85 | 0 | 1.2 | 0 | 0 | 0 |
| Development environment | 0 | 0 | 0 | 0.29 | 0 | 0.82 |
| Platform | 0.85 | 0.02 | 3.51 | 2.07 | 7.01 | 10.1 |
| Valuation | 0 | 0 | 0 | 1.57 | 2.41 | 5.38 |
| N/W infra | 0 | 0 | 0 | 0.08 | 0 | 0.08 |
| N/W-based real-time distributed control | 0 | 0 | 0 | 0 | 0 | 0 |
| N/W-based robot server | 0.43 | 0.02 | 1.74 | 2.23 | 3.91 | 5.99 |
| N/W-based distributed intelligence | 0 | 0 | 0 | 0.21 | 0 | 0.52 |
| N/W-based service | 0 | 0 | 0 | 2.02 | 2.64 | 4.47 |

Appendix C. Proportions of innovator nationalities for each technology

| **Technology** | **USPTO** | | | | **KIPO** | | | |
| --- | --- | --- | --- | --- | --- | --- | --- | --- |
|  | **US** | **KR** | **JP** | **EU** | **US** | **KR** | **JP** | **EU** |
| Wheel drive | 0.59 | 0.17 | 0.12 | 0.04 | 0 | 0.94 | 0 | 0.04 |
| Biped walking | 0.13 | 0.13 | 0.47 | 0.2 | 0.01 | 0.62 | 0.37 | 0 |
| Movement to stair or dangerous area | 0.4 | 0.2 | 0.4 | 0 | 0 | 1 | 0 | 0 |
| Manipulator | 0.66 | 0.02 | 0.12 | 0.18 | 0.03 | 0.85 | 0.04 | 0.07 |
| Robot arm | 0.04 | 0.1 | 0.74 | 0.12 | 0.06 | 0.57 | 0.33 | 0.04 |
| Haptic device | 0.92 | 0 | 0 | 0 | 0.03 | 0.93 | 0.03 | 0 |
| Robot eye or neck device | 0.68 | 0.08 | 0.16 | 0.03 | 0.02 | 0.75 | 0.21 | 0.02 |
| Joint | 0.35 | 0.07 | 0.53 | 0.03 | 0.12 | 0.76 | 0.11 | 0.01 |
| Vision recognition | 0.38 | 0.06 | 0.38 | 0.19 | 0 | 0.89 | 0.11 | 0 |
| Voice recognition | 0.08 | 0.08 | 0.83 | 0 | 0 | 0.89 | 0.11 | 0 |
| Cartography | 0.95 | 0 | 0 | 0 | 0 | 1 | 0 | 0 |
| Self-localization | 0.2 | 0.21 | 0.51 | 0.06 | 0 | 0.98 | 0.01 | 0.01 |
| Environmental recognition | 0.32 | 0.06 | 0.53 | 0.03 | 0 | 0.88 | 0.13 | 0 |
| Learning and inference | 0.6 | 0 | 0.3 | 0.03 | 0.02 | 0.91 | 0.06 | 0.02 |
| Context/semantics | 0.64 | 0 | 0.07 | 0.21 | 0 | 1 | 0 | 0 |
| Sensor fusion | 0.29 | 0.07 | 0.29 | 0.14 | 0.02 | 0.98 | 0 | 0 |
| Control architecture | 0.82 | 0.06 | 0 | 0.06 | 0.04 | 0.88 | 0.07 | 0.02 |
| Navigation control | 0.67 | 0.08 | 0.08 | 0.15 | 0 | 0.93 | 0.06 | 0 |
| Walking control | 0.08 | 0.13 | 0.75 | 0.02 | 0 | 0.72 | 0.28 | 0 |
| Manipulation | 0.68 | 0 | 0.21 | 0.08 | 0 | 1 | 0 | 0 |
| Intelligence control | 0.59 | 0.05 | 0.09 | 0.05 | 0 | 1 | 0 | 0 |
| Motion sensor or tactile sensor | 0.55 | 0.07 | 0.26 | 0.05 | 0.06 | 0.88 | 0.06 | 0 |
| Visual sensor | 0.43 | 0 | 0.47 | 0.08 | 0.07 | 0.79 | 0.14 | 0 |
| Auditory sensor | 0.73 | 0.18 | 0.09 | 0 | 0 | 0.95 | 0.05 | 0 |
| Distance sensor | 0.33 | 0.18 | 0.24 | 0.12 | 0.04 | 0.92 | 0.04 | 0 |
| Position sensor | 0.61 | 0.05 | 0.15 | 0.13 | 0.05 | 0.88 | 0.05 | 0.03 |
| Biological signal sensor | 0.5 | 0.25 | 0 | 0 | 0 | 1 | 0 | 0 |
| Olfactory sensor and taste sensor | 1 | 0 | 0 | 0 | 0 | 1 | 0 | 0 |
| Motor | 0.46 | 0.09 | 0.29 | 0.07 | 0.02 | 0.89 | 0.09 | 0.01 |
| Artificial muscle | 0.42 | 0 | 0.32 | 0.05 | 0 | 1 | 0 | 0 |
| Decelerator | 0 | 0.14 | 0.71 | 0.14 | 0.01 | 0.64 | 0.29 | 0.03 |
| Fuel cell | 0.25 | 0.5 | 0.13 | 0 | 0 | 1 | 0 | 0 |
| Ion battery | 0 | 0 | 0 | 0 | 0 | 1 | 0 | 0 |
| Solar fuel | 0.86 | 0 | 0.14 | 0 | 0 | 1 | 0 | 0 |
| SoC (System on Chip) | 0 | 0 | 0 | 0 | 0 | 1 | 0 | 0 |
| Fusion module | 0 | 0 | 0 | 0 | 0 | 1 | 0 | 0 |
| Distributed object | 1 | 0 | 0 | 0 | 0 | 1 | 0 | 0 |
| Development environment | 1 | 0 | 0 | 0 | 0 | 1 | 0 | 0 |
| Platform | 0.75 | 0 | 0 | 0 | 0.03 | 0.9 | 0.03 | 0.04 |
| Valuation | 0 | 0 | 0 | 0 | 0.04 | 0.81 | 0.06 | 0.08 |
| N/W infra | 0 | 0 | 0 | 0 | 0 | 0 | 0 | 0 |
| N/W-based real-time distributed control | 0 | 0 | 0 | 0 | 0 | 0 | 0 | 0 |
| N/W-based robot server | 0.43 | 0.36 | 0.21 | 0 | 0 | 0.98 | 0.02 | 0 |
| N/W-based distributed intelligence | 0 | 0 | 0 | 0 | 0 | 1 | 0 | 0 |
| N/W-based service | 0 | 0 | 0 | 0 | 0 | 1 | 0 | 0 |

Appendix D. Average interval between application and registration dates and average number of claims for each technology

| Technologies | USPTO | | KIPO | |
| --- | --- | --- | --- | --- |
|  | Period between application and registration dates [days] | Number of claims | Period between application and registration dates [days] | Number of claims |
| Wheel drive | 382 | 21 | 525 | 8 |
| Biped walking | 385 | 13 | 385 | 12 |
| Movement to stair or dangerous area | 413 | 16 | 504 | 9 |
| Manipulator | 385 | 23 | 501 | 10 |
| Robot arm | 384 | 14 | 452 | 8 |
| Haptic device | 353 | 24 | 501 | 10 |
| Robot eye or neck device | 290 | 21 | 466 | 7 |
| Joint | 470 | 15 | 467 | 10 |
| Vision recognition | 497 | 22 | 466 | 11 |
| Voice recognition | 370 | 15 | 449 | 9 |
| Cartography | 374 | 26 | 343 | 12 |
| Self-localization | 424 | 16 | 518 | 9 |
| Environmental recognition | 383 | 17 | 492 | 12 |
| Learning and inference | 349 | 19 | 501 | 9 |
| Context/semantics | 483 | 22 | 534 | 9 |
| Sensor fusion | 350 | 18 | 521 | 12 |
| Control architecture | 292 | 20 | 498 | 9 |
| Navigation control | 316 | 20 | 514 | 8 |
| Walking control | 359 | 13 | 425 | 12 |
| Manipulation | 436 | 24 | 571 | 8 |
| Intelligence control | 417 | 17 | 523 | 11 |
| Motion sensor or tactile sensor | 429 | 20 | 489 | 12 |
| Visual sensor | 287 | 20 | 443 | 10 |
| Auditory sensor | 309 | 26 | 441 | 13 |
| Distance sensor | 343 | 25 | 503 | 9 |
| Position sensor | 413 | 24 | 504 | 9 |
| Biological signal sensor | 352 | 25 | 519 | 24 |
| Olfactory sensor and taste sensor | 1721 | 30 | 549 | 9 |
| Motor | 441 | 17 | 521 | 6 |
| Artificial muscle | 404 | 16 | 530 | 10 |
| Decelerator | 359 | 13 | 437 | 8 |
| Fuel cell | 488 | 26 | 553 | 13 |
| Ion battery | 0 | 0 | 583 | 4 |
| Solar fuel | 296 | 21 | 522 | 11 |
| SoC (System on Chip) | 0 | 0 | 187 | 24 |
| Fusion module | 0 | 0 | 556 | 10 |
| Distributed object | 258 | 1 | 554 | 17 |
| Development environment | 554 | 20 | 516 | 10 |
| Platform | 354 | 17 | 506 | 12 |
| Valuation | 0 | 0 | 494 | 12 |
| N/W infra | 0 | 0 | 208 | 37 |
| N/W-based real-time distributed control | 0 | 0 | 0 | 0 |
| N/W-based robot server | 384 | 19 | 474 | 13 |
| N/W-based distributed intelligence | 0 | 0 | 556 | 13 |
| N/W-based service | 0 | 0 | 496 | 13 |

Appendix E. QAP results: USPTO co-occurrence matrix and coefficient of independent variables

| Source of Independent Matrix | Independent Matrix | Standardized coefficient | P-value | Standard error |
| --- | --- | --- | --- | --- |
| USPTO | Absolute difference of degree centralities | 0.52*** | 0.001 | 0.1 |
| USPTO | Sum of degree centralities | 0.35 | 0.258 | 0.51 |
| USPTO | Absolute difference of betweenness centralities | -0.03 | 0.297 | 0.09 |
| USPTO | Sum of betweenness centralities | -0.08 | 0.255 | 0.17 |
| USPTO | Absolute difference of eigenvector centralities | -0.73*** | 0.001 | 0.02 |
| USPTO | Sum of eigenvector centralities | 0.52*** | 0.001 | 0.02 |
| USPTO | Absolute difference of percentages of innovators of EU nationality | 0 | 0.958 | 0.21 |
| USPTO | Sum of percentages of innovators of EU nationality | 0 | 0.985 | 0.23 |
| USPTO | Absolute difference of percentages of innovators of JP nationality | 0.09* | 0.059 | 0.61 |
| USPTO | Sum of percentages of innovators of JP nationality | -0.17* | 0.097 | 1.28 |
| USPTO | Absolute difference of percentages of innovators of KR nationality | -0.01 | 0.426 | 1.06 |
| USPTO | Sum of percentages of innovators of KR nationality | -0.06 | 0.125 | 1.37 |
| USPTO | Absolute difference of percentages of innovators of US nationality | -0.11** | 0.027 | 0.54 |
| USPTO | Sum of percentages of innovators of US nationality | -0.03 | 0.39 | 1.22 |
| USPTO | Overlap in IPC | -0.03 | 0.257 | 0 |
| USPTO | Overlap in innovator | 0.38** | 0.001 | 0 |
| USPTO | Absolute difference of periods between application and registration dates | -0.28** | 0.038 | 0 |
| USPTO | Sum of periods between application and registration dates | 0.29** | 0.034 | 0 |
| USPTO | Absolute difference of numbers of claims | -0.06* | 0.086 | 0.02 |
| USPTO | Sum of number of claims | -0.03 | 0.391 | 0.05 |
| KIPO | Absolute difference of degree centralities | 0.1 | 0.229 | 0.12 |
| KIPO | Sum of degree centralities | -0.7* | 0.067 | 0.49 |
| KIPO | Absolute difference of betweenness centralities | -0.11** | 0.037 | 0.06 |
| KIPO | Sum of betweenness centralities | -0.02 | 0.433 | 0.08 |
| KIPO | Absolute difference of eigenvector centralities | -0.21* | 0.07 | 0.02 |
| KIPO | Sum of eigenvector centralities | 0.47** | 0.035 | 0.03 |
| KIPO | Absolute difference of percentages of innovators of EU nationality | -0.06* | 0.098 | 5.91 |
| KIPO | Sum of percentages of innovators of EU nationality | -0.03** | 0.044 | 0.29 |
| KIPO | Absolute difference of percentages of innovators of JP nationality | -0.02 | 0.441 | 2.38 |
| KIPO | Sum of percentages of innovators of JP nationality | -0.11* | 0.072 | 4.04 |
| KIPO | Absolute difference of percentages of innovators of KR nationality | 0.06 | 0.271 | 1.87 |
| KIPO | Sum of percentages of innovators of KR nationality | -0.02 | 0.182 | 4.63 |
| KIPO | Absolute difference of percentages of innovators of US nationality | -0.07 | 0.111 | 4.39 |
| KIPO | Sum of percentage of innovators of US nationality | 0.07 | 0.125 | 5.69 |
| KIPO | Overlap in IPC | -0.03 | 0.332 | 0 |
| KIPO | Overlap in innovator | 0.01 | 0.42 | 0 |
| KIPO | Absolute difference of periods between application and registration dates | -0.07* | 0.073 | 0 |
| KIPO | Sum of periods between application and registration dates | -0.16** | 0.016 | 0 |
| KIPO | Absolute difference of numbers of claims | -0.01 | 0.398 | 0.04 |
| KIPO | Sum of number of claims | 0.01 | 0.431 | 0.05 |
|  | Intercept | 0 | 0 | 0 |

* p<0.1 ,** p<0.05 , *** p<0.01.

Appendix F. QAP results: KIPO co-occurrence matrix and coefficient of independent variables

| Source of Independent Matrix | Independent Matrix | Standardized Coefficient | P-value | Standard Error |
| --- | --- | --- | --- | --- |
| USPTO | Absolute difference of degree centralities | 0.06 | 0.293 | 0.21 |
| USPTO | Sum of degree centralities | -0.43 | 0.178 | 1.09 |
| USPTO | Absolute difference of betweenness centralities | 0.08 | 0.091 | 0.19 |
| USPTO | Sum of betweenness centralities | 0.02 | 0.441 | 0.36 |
| USPTO | Absolute difference of eigenvector centralities | -0.12 | 0.107 | 0.04 |
| USPTO | Sum of eigenvector centralities | 0 | 0.435 | 0.03 |
| USPTO | Absolute difference of percentages of innovators of EU nationality | 0.03*** | 0.001 | 1.32 |
| USPTO | Sum of percentages of innovators of EU nationality | 0.03*** | 0.001 | 1.44 |
| USPTO | Absolute difference of percentages of innovators of JP nationality | 0.08** | 0.031 | 1.18 |
| USPTO | Sum of percentages of innovators of JP nationality | 0.13 | 0.113 | 2.96 |
| USPTO | Absolute difference of percentages of innovators of KR nationality | 0.04 | 0.197 | 2.23 |
| USPTO | Sum of percentages of innovators of KR nationality | 0 | 0.482 | 2.92 |
| USPTO | Absolute difference of percentages of innovators of US nationality | -0.06* | 0.092 | 1.07 |
| USPTO | Sum of percentages of innovators of US nationality | 0.14* | 0.075 | 2.83 |
| USPTO | Overlap in IPC | -0.06* | 0.09 | 0 |
| USPTO | Overlap in innovator | 0 | 0.434 | 0.01 |
| USPTO | Absolute difference of periods between application and registration dates | 0 | 0.506 | 0 |
| USPTO | Sum of periods between application and registration dates | 0.06 | 0.273 | 0 |
| USPTO | Absolute difference of numbers of claims | -0.03 | 0.207 | 0.05 |
| USPTO | Sum of number of claims | 0.01 | 0.49 | 0.1 |
| KIPO | Absolute difference of degree centralities | 0.45*** | 0.001 | 0.26 |
| KIPO | Sum of degree centralities | 0.29 | 0.2 | 1.06 |
| KIPO | Absolute difference of betweenness centralities | -0.03 | 0.236 | 0.11 |
| KIPO | Sum of betweenness centralities | -0.05 | 0.218 | 0.17 |
| KIPO | Absolute difference of eigenvector centralities | -1.04*** | 0.001 | 0.04 |
| KIPO | Sum of eigenvector centralities | 1.18*** | 0.001 | 0.07 |
| KIPO | Absolute difference of percentages of innovators of EU nationality | 0.01 | 0.31 | 12.44 |
| KIPO | Sum of percentages of innovators of EU nationality | -0.03 | 0.998 | 1.96 |
| KIPO | Absolute difference of percentages of innovators of JP nationality | -0.33*** | 0.001 | 4.71 |
| KIPO | Sum of percentages of innovators of JP nationality | 0.07 | 0.238 | 8.35 |
| KIPO | Absolute difference of percentages of innovators of KR nationality | 0.24*** | 0.002 | 3.89 |
| KIPO | Sum of percentages of innovators of KR nationality | 0.01 | 0.27 | 10.52 |
| KIPO | Absolute difference of percentages of innovators of US nationality | -0.2*** | 0.001 | 8.81 |
| KIPO | Sum of percentages of innovators of US nationality | 0.21*** | 0.003 | 13.2 |
| KIPO | Overlap in IPC | 0.01 | 0.443 | 0 |
| KIPO | Overlap in innovator | 0.41*** | 0.001 | 0 |
| KIPO | Absolute difference of periods between application and registration dates | -0.03 | 0.209 | 0.01 |
| KIPO | Sum of periods between application and registration dates | 0.02 | 0.416 | 0.01 |
| KIPO | Absolute difference of numbers of claims | -0.18*** | 0.001 | 0.09 |
| KIPO | Sum of number of claims | 0.26*** | 0.001 | 0.12 |
|  | Intercept | 0 | 0 | 0 |

* p<0.1, ** p< 0.05, *** p<0.01.
